# miR-27a-3p suppresses tumor metastasis and VM by down-regulating VE-cadherin expression and inhibiting EMT: an essential role for Twist-1 in HCC

**DOI:** 10.1038/srep23091

**Published:** 2016-03-16

**Authors:** Nan Zhao, Huizhi Sun, Baocun Sun, Dongwang Zhu, Xiulan Zhao, Yong Wang, Qiang Gu, Xueyi Dong, Fang Liu, Yanhui Zhang, Xiao Li

**Affiliations:** 1 Department of Pathology, Tianjin Medical University, Tianjin 300070, China; 2 Department of Pathology, General Hospital of Tianjin Medical University, Tianjin 300052, China; 3 Department of Pathology, Cancer Hospital of Tianjin Medical University, Tianjin 300060, China; 4 Stomatological Hospital, Tianjin Medical University, Tianjin 300070, China

## Abstract

Twist-1 and miRNAs have been reported to be associated with tumor metastasis and angiogenesis. However, the relationship between Twist-1 and miRNAs and the function of miRNAs remain largely undefined. We aimed to reveal the Twist-1-related miRNA expression profile and to determine whether Twist-1 functions in tumor metastasis and vasculogenic mimicry (VM) by regulating miRNA expression in hepatocellular carcinoma (HCC). Results showed that the expression of miR-27a-3p was consistently down-regulated in HCC cell lines and tissue samples displaying high expression of Twist-1. Both loss- and gain-of-function assays revealed suppressive effects of miR-27a-3p. Low miR-27a-3p expression was significantly associated with early metastasis in HCC. Subsequent investigations revealed that miR-27a-3p mediated the inhibition of epithelial–mesenchymal transition (EMT). Additional experiments showed that VE-cadherin is a direct target of miR-27a-3p and further demonstrated the critical role of miR-27a-3p in suppressing tumor metastasis and VM. Conclusions: Twist-1 up-regulation in HepG2 cells resulted in the differential expression of 18 miRNAs. Among them, miR-27a-3p deregulation contributed to VM and metastasis. The miR-27a-3p-mediated down-regulation of VE-cadherin and inhibition of EMT may be essential for Twist-1 to induce tumor metastasis and VM. Our findings highlight the importance of miR-27a-3p and suggest a promising new strategy for anti-HCC therapy.

Twist-1, an evolutionarily conserved transcription factor in the basic helix-loop-helix family, was originally reported to act as a master regulator of embryonic morphogenesis^1^. Recent studies revealed that Twist-1-induced epithelial–mesenchymal transition (EMT) enhances behaviors of HCC including invasion, metastasis and vasculogenic mimicry (VM) formation^1^,^2^,^3^. In recent years, aberrant regulation of microRNAs (miRNAs) has been proposed to be associated with Twist-1^4^,^5^.

miRNAs are a class of diverse, small, non-coding RNAs of 21–25 nucleotides in length that target mRNAs by directly binding to their 3′-untranslated region (3′-UTR) to cause their degradation or translational suppression. miRNAs can function as tumor suppressors or promoters, depending on whether they specifically target oncogenes or tumor suppressor genes, respectively^6^,^7^. Emerging evidence suggests that deregulation of miRNAs may contribute to tumor metastasis and angiogenesis^8^,^9^,^10^. Specifically, several studies have focused on the effect of miRNAs on the metastasis and angiogenesis of hepatocellular carcinoma (HCC) cells^7^. miR-34a, miR-29b, and miR-214 inhibit and miR-10a, miR-331–3p, and miR-221 promote metastasis and angiogenesis in HCC cells^11^.

Given the potential role of miRNAs in HCC, their expression was profiled in HepG2 cells stably transfected with an empty vector (HepG2-vector) or a Twist-1 expression vector (HepG2-Twist-1) using microarray and ChIP-seq technology (the protein used for ChIP was Twist-1). The differentially expressed miRNAs, as verified by quantitative real-time PCR (qRT-PCR), were subjected to gene ontology (GO) analysis. Furthermore, miR-27a-3p was identified as a tumor suppressive miRNA in human HCC that acts by repressing vascular endothelial (VE)-cadherin and mesenchymal markers of EMT, which have previously been reported to be important for the function of Twist-1 in tumor metastasis and VM. These results provide additional evidence for a crucial role of miRNAs in Twist-1-mediated HCC metastasis, invasion, and VM.

The expression of miR-27a-3p varies from one cancer type to another; thus, the functions of miR-27a-3p are very diverse among different malignancies. Therefore, the functional role of miR-27a-3p is extremely complex: it can perform tumor suppressive or oncogenic functions in different tumor types. The phenotype caused by aberrant miR-27a-3p expression appears to be strongly dependent on its endogenous expression level. For instance, miR-27a-3p was highly over-expressed in invasive clear cell renal cell carcinoma and in laryngeal carcinoma, and in these cancer types, miR-27a-3p expression correlated with metastasis and aggressiveness^12^,^13^. In some cancer types, such as esophageal squamous cell carcinoma (ESCC) and colorectal cancer, miR-27a-3p is significantly down-regulated and functions as a tumor suppressor^14^,^15^. However, although the expression of miR-27a-3p and its functions were extensively studied and well defined in many cancer types, the role of miR-27a-3p in human HCC and its association with Twist-1 remain unknown.

In this study, we analyzed the correlation between Twist-1 expression and miR-27a-3p expression in HCC cells and tissue samples. Gain- and loss-of-function assays were performed to determine the effect of miR-27a-3p on tumor cell behaviors. Additionally, we used xenograft mice models to investigate the role of miR-27a-3p in tumor metastasis and VM formation *in vivo*. Finally, we explored the molecular mechanisms underlying the function of miR-27a-3p and its potential targets.

## Materials and Methods

### Cell culture and transfection

The following cell lines were obtained from the American Type Culture Collection and from Zhongshan Hospital Affiliated to Fudan University (Shanghai, China) in 2014: H7402, Bel7402, SMMC, HepG2, PLC, L02 and 293T cell lines were cultured in Dulbecco’s modified Eagle’s medium (HyClone) supplemented with 10% fetal bovine serum (FBS, HyClone). Plasmid vectors were transfected into the cells with polyethylenimine (PEI) (PolyScience, Inc., Cat#23966).

### miRNA microarray and chromatin immunoprecipitation (ChIP)-seq analyses

Samples of HepG2-vector and HepG2-Twist-1 cells were collected. Data from miRNA microarray and ChIP-seq assays (using a polyclonal antibody targeting Twist-1) were used to analyze the sequences of miRNAs that appeared to directly bind to the Twist-1 protein. The analyses, including GO analysis, pathway analysis, and microRNA-gene net work analysis, were completed by Gminix Biotechnology Company in Shanghai.

### Chromatin immunoprecipitation (ChIP)-qPCR

Chromatin immunoprecipitation (ChIP) assay was performed as previously described^16^. Real-time PCR was conducted by SYBR Green-based detection method (Applied Biosystems) using equal amounts of ChIP and diluted input DNAs. Antibodies used for the ChIP assays included anti-Twist-1 (SC-15393 X, Santa Cruz, USA) and irrelevant IgG control antibody (AB171870, Abcam, UK).

### Patient samples

Forty-two human HCC and thirty-five noncancerous liver tissues were collected from patients undergoing resection of HCC at the Tumor Tissue Bank of Tianjin Cancer Hospital. Pathologists verified the diagnoses of these HCC samples. Detailed pathologic and clinical data were collected for all samples. Informed consent was obtained from each patient.

All of the experimental protocols were conducted in accordance with the approved guidelines and were approved by the Ethics Committee of Tianjin Cancer Hospital.

### Plasmids

The miExpress™ Precursor miRNA Expression Clone miR-27a-3p (Catalog No.: HmiR0297-MR04) (referred to as miR-27a), the miRNA Control Vector (Catalog No.: CmiR0001-MR04) (revered to as miR-Con), the miArrest™ miRNA Inhibitor Expression Clone miR-27a-3p (Catalog No.: HmiR-AN0359-AM02) (referred to as miR-inhibitor-27a), and the miRNA inhibitor control (Catalog No.: CmiR-AN0001-AM02) (referred to as miR-inhibitor-Con) were constructed by GeneCopoeia Inc. The pcDNA3-Twist-1 plasmid (defined as pcDNA-Twi) was obtained by cDNA library subcloning and was verified by sequencing. The small interfering RNA (siRNA) kit (pGP-Twist-1-shRNA) (defined as sh-RNA-Twi) was purchased from GenePharma (Shanghai, China).

### Immunofluorescence

Cells were adhered to coverslips and were allowed to grow to 50–60% confluence. Then, the cells were fixed for 10 min in cold methanol and blocked with fetal calf serum. The cells were incubated with primary and secondary antibodies. FITC- and TRITC-conjugated mouse and rabbit IgG antibodies (Santa Cruz) were used to label the cells for immunofluorescence assays. After immunolabeling, the cells were washed, stained with DAPI (Sigma), mounted, and viewed under a fluorescence microscope (Nikon, Japan).

### Wound healing and cell invasion assays

Via wound healing assays, cell motility was assessed by measuring the movement of cells into a scratch. The speed of wound closure was monitored at 24 h and 48 h by measuring the ratio of the width of the wound at these time points to the distance of the wound at 0 h. Cell invasion assays were performed using transwell cell culture inserts (Invitrogen). Transfected cells were maintained for 48 h and allowed to migrate for an additional 24 h. The migrated cells were stained with crystal violet solution, and their absorbance was determined at 570 nm. Each experiment was performed in triplicate.

### Cell adhesion assay

Matrigel was purchased from BD, and a working concentration of this matrix was used according to specific instructions. Confluent cultures of tumor cells were briefly trypsinized, plated on the Matrigel-coated insert of 24-well culture plates and allowed to attach for 2 h in standard culture medium. Then, the medium liquid was discarded, and the cells were gently washed once with PBS. The cells were stained with crystal violet solution, washed three times with PBS, and dissolved using ethanol. The optical density and the percentage of viable cells were determined based on the absorbance at 570 nm (A570). Each experiment was performed in triplicate, and the mean values ± SE are presented.

### 3D VM formation

Matrigel (Collaborative Biomedical) was thawed at 4 °C, and 200 μl of Matrigel was rapidly added to each well of a 24-well plate, allowed to solidify for one hour at room temperature, and placed at 37 °C in a humidified 5% CO_2_ incubator for 30 minutes. Tumor cells were transfected for 24 h, seeded in complete medium in Matrigel-coated wells, and incubated at 37 °C for 48 h.

### RNA extraction

Total RNA was extracted using Trizol reagent (Tiangen Biotech), and miRNA samples were obtained using the miRcute miRNA isolation kit (DP501) (Tiangen Biotech).

### Semiquantitative RT-PCR and qRT-PCR

RT-PCR and qRT-PCR were performed as previously described^17^,^18^ using the primers listed in Table S1. PCR was conducted using a 7500HT Real-Time PCR System (Applied Biosystems, Foster City, CA, USA). U6 or GAPDH was used as an endogenous internal control, and the fold-changes in expression were calculated via relative quantification (2^−∆∆Ct^)^19^.

### Western blot

Cells were lysed and then transferred to polyvinylidene difluoride (PVDF) membranes (Millipore). The membranes were blocked and incubated with primary antibodies (Table S2) for 1 h at room temperature with agitation, followed by incubation with secondary antibodies (1:2000; Santa Cruz). Then, the membranes were developed using an enhanced chemiluminescence (ECL) detection kit (Amersham Pharmacia Biotech, Piscataway, NJ, USA). A rabbit polyclonal β-actin antibody (sc1616-R, 1:200; Santa Cruz) was used as a protein loading control. The intensity of the protein bands was determined via densitometry using Image J system.

### Gelatin zymography

All media were collected and subjected to SDS-PAGE using 10% polyacrylamide gels containing 0.01% w/v gelatin. After electrophoresis, the gels were equilibrated in 2.5% Triton X-100 and incubated in 50 mM Tris-HCl (pH 7.5), 10 mM CaCl2, 150 mM NaCl, 1 mM ZnCl2, and 0.02% NaN3 for 40 h at 37 °C. Subsequently, the gels were stained with Coomassie R250 and de-stained until the wash solution became clear and clear bands associated with MMP activity appeared in the gels. The intensity of the bands were determined via densitometry using Image J system.

### Luciferase reporter assay

293T cells were transiently co-transfected with miR-27a-3p, miR-control, miR-27a-3p-inhibitor, miR-inhibitor-control, pGluc/SEAP-CDH5-3′ UTR-wt, pGluc/SEAP-CDH5-3′ UTR-mt, or pGluc/SEAP-vector. After 60 hours, the cell culture medium was collected. Secrete-Pair^TM^ Dual Luminescence Assay Kits (GeneCopoeia^TM^) were used to examine these samples. The SEAP signal was used as a normalization control; the luminescence intensities of GLuc and SEAP were detected using a Synergy™ Mx Multi-Mode Microplate Reader (BioTek, USA). The normalized GLuc activity (GLuc/SEAP ratio) of all samples was compared. The results were obtained from three independent experiments performed in duplicate.

### Animal study

For the subcutaneous xenograft model, SMMC cells (5 × 10^6^) and HepG2 cells (5 × 10^6^) (stably transfected with miR-27a-3p, the miR-27a-3p inhibitor, and the corresponding control vector) were suspended in 100 μl of PBS and then subcutaneously injected into the upper right flank region of female BALB/c-nu/nu mice at 3–4 weeks of age. After 4–5 weeks, the mice were scarified, and tumors were dissected, fixed in formalin, and embedded in paraffin.

All of the experimental protocols were conducted in accordance with our Institutional Animal Care and Use Committee (IACUC) guidelines and were approved by the Tianjin Medical University IACUC committee.

### IHC and endomucin/periodic acid-schiff (PAS) double staining analysis of MVD

The sections were preheated in a microwave, blocked, and incubated in a series of antibodies (Table S2). The staining systems used in this study were PicTure PV6000 (Zhongshan Chemical Co., Beijing, China) and Elivision Plus (Zhongshan Chemical Co., Beijing, China). After IHC staining for endomucin was performed, the sections were washed with running water for 5 minutes and incubated with periodic acid for 8 min and schiff for 15 min. All of the sections were counterstained with hematoxylin, dehydrated, and mounted. Phosphate-buffered saline was used in place of the primary antibodies for the negative control. The results were quantified according to the method described by Bittner *et al.*

### Statistical analysis

All data were evaluated using SPSS 22 (SPSS Inc., Chicago, IL, USA). All statistical analyses were performed using ANOVA or a two-tailed Student’s t-test to compare the data. Survival curves were calculated using the Kaplan-Meier method. Differences were considered significant at p < 0.05. Significant differences between groups are labeled with an asterisk in the figures.

## Results

### miRNA microarray and ChIP-seq analyses revealed the relationship of miR-27a-3p with Twist-1 in HepG2 cells

**Figure 1:**
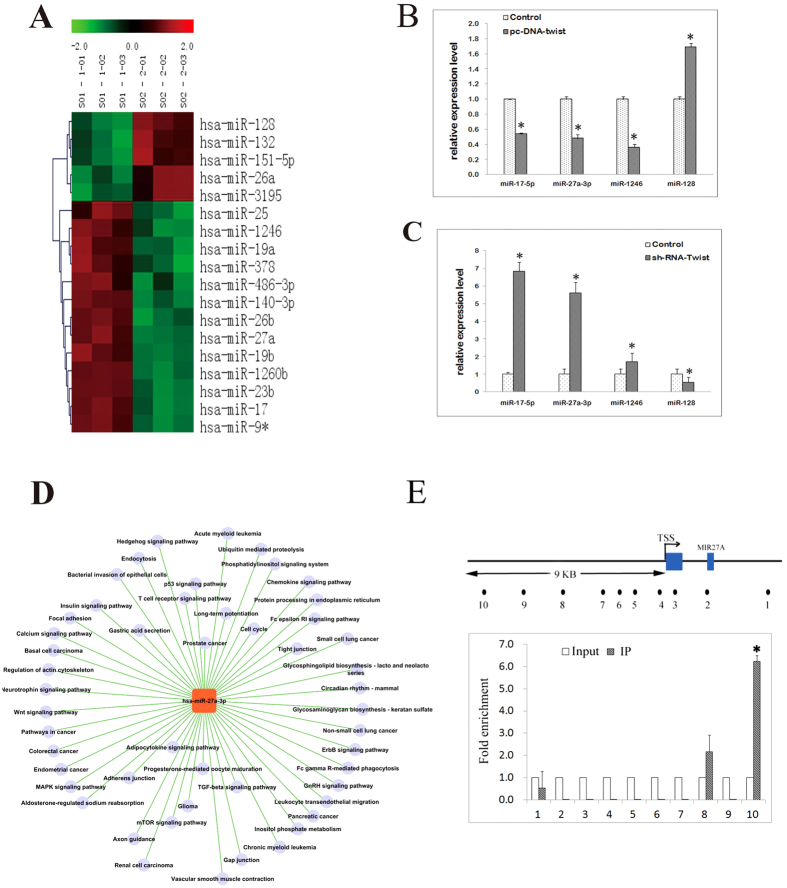
Twist-1 over-expression down-regulates the expression of miR-27a-3p. (**A**) Profile of miRNAs that were differentially expressed between HepG2-Twist-1 cells and HepG2-vector cells. The results were obtained from three paired samples. Both down-regulated (green) and up-regulated (red) miRNAs were identified in HepG2-Twist-1 cells compared to HepG2-vector cells. (**B**,**C**) Validation of the microarray data via real-time RT-PCR. Triplicate assays were performed for each RNA sample, and the relative quantity of each miRNA was normalized to the quantity of U6 snRNA. Statistically significant differences are indicated by *p < 0.01. (**D**) Microarray-based GO analysis revealed the roles of miR-27a-3p in HCC. (**E**) ChIP was performed on HepG2 cells transfected with pcDNA-Twi. The precipitated chromatin was PCR-amplified with the use of specific primers in the Pre-mir-27a promoter (Supplementary Table S3) as indicated by black dots. Bar graphs show fold enrichment of Twist-1 binding of MIR27A Transcription Start Site (TSS) region. Relative enrichment compared to irrelative antibody control is shown. The mean ± s.e.m of three determinations is shown. Data represent the mean ± SD of three experiments.

miRNA microarray analysis identified 357 miRNAs that were differentially expressed between HepG2-Twist-1 cells and HepG2-vector cells. Among these miRNAs, 18 were validated to be significantly differentially expressed between HepG2-vector and HepG2-Twist-1 cells (*p* < 0.05) (Table 1) (Fig. 1A). Functional analysis of these miRNAs via ChIP-seq (using polyclonal antibody targeting Twist-1) revealed that miR-17-5p, miR-27a-3p, miR-1246, and miR-128 may directly bind to the Twist-1 protein. As shown in Fig. 1B,C, the levels of miR-17-5p, miR-27a-3p, and miR-1246 were down-regulated in HepG2 cells over-expressing Twist-1 and were up-regulated in HepG2 cells displaying reduced expression of Twist-1; miR-128 showed the opposite expression pattern. These findings were in agreement with the results of hybridized microarray analysis.

The GO network of differentially expressed miRNAs showed that miR-27a-3p was involved in 50 GO annotations (Fig. 1D), including the Wnt signaling pathway, actin cytoskeleton regulation, the mitogen-activated protein kinase (MAPK) signaling pathway, vascular smooth muscle contraction, and the transforming growth factor beta (TGFβ) signaling pathway, all of which are important pathways in VM and EMT. These Go analysis suggested that miR-27a-3p might be of great importance to VM and EMT. Therefore, miR-27a-3p was chosen for further studies.

In order to further clarify regulation mechanism of miR-27a-3p by Twist-1, we prove it by using Chromatin immunoprecipitation (ChIP)-qPCR. Primers used in this experiment were targeted to Twist-1 binding elements in promoter regions of MIR-27A (Primer sequences are listed in Table S3). Ten pairs of primers were designed. Analysis of qRT-PCR revealed that only the primer 10 showed high amplification efficiency (Fig. 1E), which suggested that Twist-1 could directly bind to the promoter region of miR-27a-3p and regulate its expression.

### miR-27a-3p expression was inversely associated with Twist-1 expression in HCC tissues and cell lines, and miR-27a-3p down-regulation is associated with metastasis

**Figure 2:**
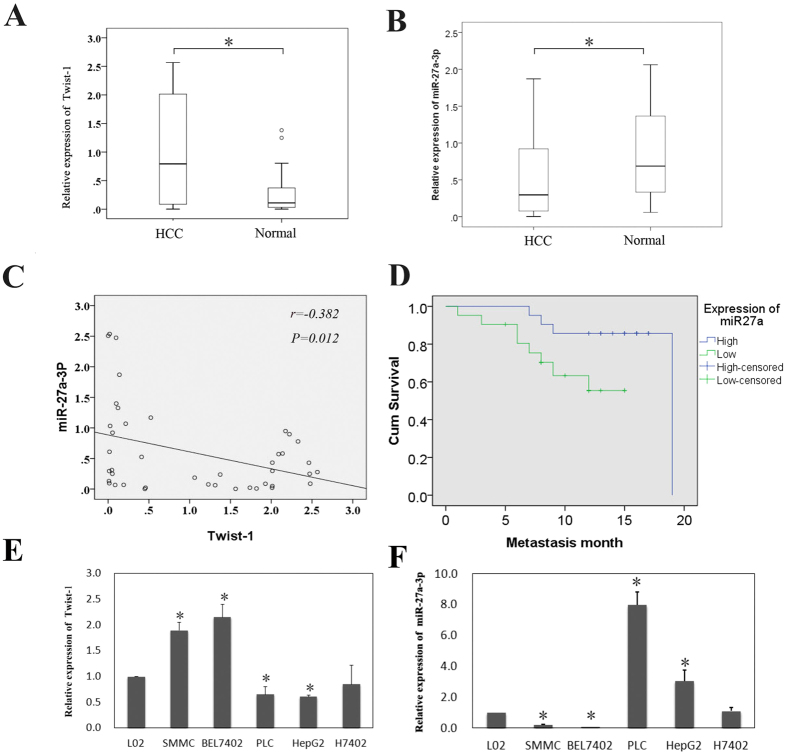
Relationship between miR-27a-3p and Twist-1 expression in HCC tissues and cell lines. (**A**) A box-and-whiskers plot showed that Twist-1 expression in HCC tissues was significantly higher than that in noncancerous tissues (*p < 0.05). (**B**) The box-and-whiskers plot directly showed that miR-27a-3p expression was significantly lower in HCC tissues than in noncancerous tissues (*p < 0.05). (**C**) Pearson correlation analysis showed that miR-27a-3p expression inversely correlated with Twist-1 expression in 42 HCC samples. (**D**) Kaplan-Meier survival curves of patients with HCC indicated that low miR-27a-3p expression was significantly associated with early metastasis of HCC. (**E**) Twist-1 expression in various HCC cell lines (*p < 0.05). (**F**) miR-27a-3p expression in various HCC cell lines (*p < 0.05).

To identify the correlation between miR-27a-3p and Twist-1 expression in HCC patients, qRT-PCR analysis was performed on 42 human HCC tissues and 35 noncancerous liver tissues. The relevant characteristics of the HCC samples are shown in Table 2. The box-and-whiskers plot shows that Twist-1 expression in HCC tissues was significantly higher than that in the noncancerous tissues (Fig. 2A, *p* < 0.05). Conversely, miR-27a-3p expression was significantly lower in HCC tissues than in noncancerous tissues (Fig. 2B, p < 0.05). Pearson correlation analysis showed that miR-27a-3p expression inversely correlated with Twist-1 expression in the 42 HCC samples (r = −0.382, p = 0.012) (Fig. 2C). In addition, Kaplan-Meier survival curves of patients with HCC indicated that low miR-27a-3p expression was significantly associated with early metastasis of HCC (Fig. 2D).

Additionally, the endogenous expression of miR-27a-3p and Twist-1 was investigated in six different liver cell lines, including five HCC cell lines (H7402, Bel7402, SMMC, HepG2 and PLC) and one normal liver cell line (L02), which served as the control. These data demonstrated that miR-27a-3p expression negatively correlated with Twist-1expression (Fig. 2E,F).

Taken together, these results indicated that Twist-1 was highly expressed in HCC but that miR-27a-3p tended to display reduced expression. Moreover, miR-27a-3p down-regulation was associated with metastasis. Further experiments exploring the role of miR-27a-3p in Twist-1-mediated VM and metastasis were warranted. According to the above results, Bel7402, SMMC, HepG2 and PLC cells were chosen for further studies.

### miR-27a-3p inhibits HCC cell metastasis, invasion, and VM formation *in vitro*

**Figure 3:**
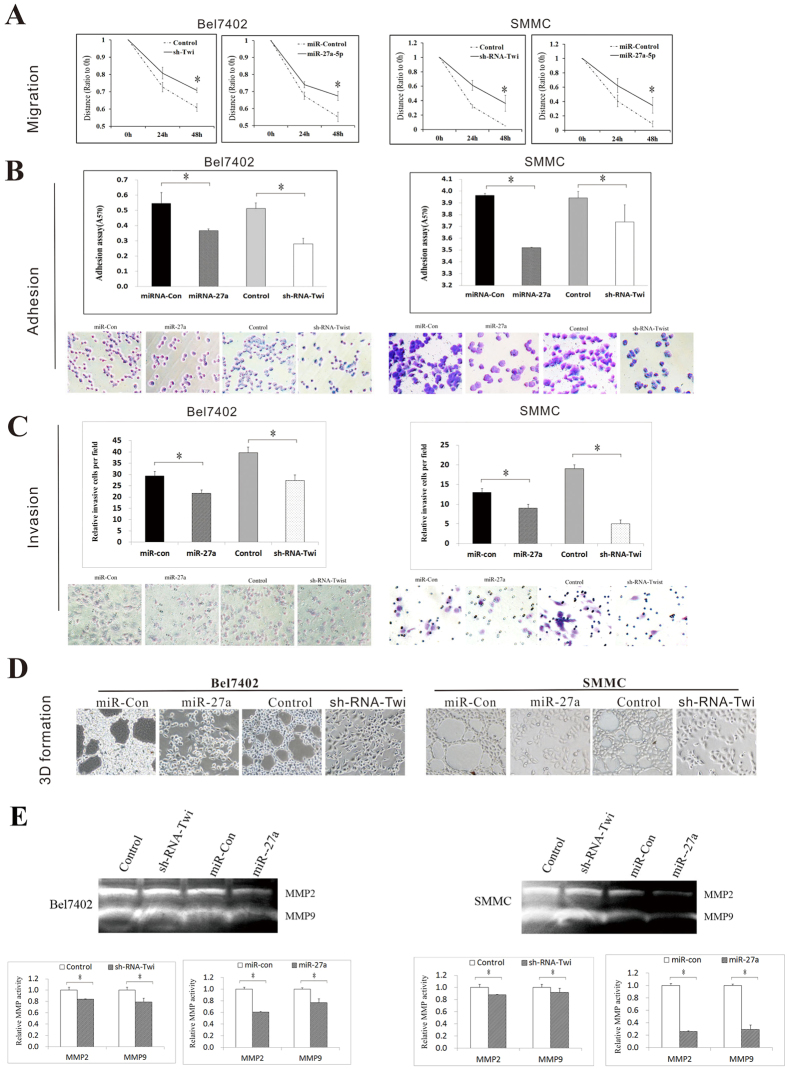
Ectopic expression of miR-27a-3p inhibited aggressive phenotypes and VM formation. Bel7402 and SMMC cells were transfected with miExpress™ Precursor miRNA Expression Clone miR-27a-3p (referred to as miR-27a) orpGP-Twist-1-shRNA (referred to as sh-RNA-Twi). The effects of miR-27a and sh-RNA-Twi on cell migration, invasion and adhesion and capillary tube formation were evaluated via (**A**) a wound healing assay, (**B**) a cell adhesion assay, (**C**) a transwell invasion assay, (**D**) a 3D VM formation assay, and (**E**) gelatin zymography.

VM formation has been associated with cell migration, invasion, and adhesion and with capillary tube formation. We transfected the miExpress™ Precursor miRNA Expression Clone miR-27a-3p (named as miR-27a in Fig. 3) into Bel7402 and SMMC cells, which display low endogenous expression of miR-27a-3p. Wound healing, cell adhesion, transwell invasion, 3D VM formation assays and gelatin zymography were performed to evaluate the effects of miR-27a-3p on cellular behaviors and VM formation. Alternatively, Bel7402 and SMMC cells, which display high endogenous expression of Twist-1, were transfected with pGP-Twist-1-shRNA (denoted as sh-RNA-Twi in Fig. 3). Then, changes in cellular behavior were evaluated to verify the role of Twist-1 in VM formation to confirm the correlation of miR-27a-3p and Twist-1 from another perspective.

We assessed the role of miR-27a-3p in regulating HCC cell migration using wound healing assays (Fig. 3A). The relative distance of cell migration was reduced by approximately 12% in Bel7402 cells and 26% in SMMC cells due to over-expression of miR-27a-3p; this result indicated that miR-27a-3p suppresses cell migration. The relative distance of cell migration was reduced by approximately 10% in Bel7402 cells and 21% in SMMC cells due to transfection with pGP-Twist-1-shRNA; these data indicated that high expression of miR-27a-3p exerts a similar effect on cell migration to down-regulation of Twist-1. Moreover, cell adhesion and transwell assays showed that miR-27a-3p expression significantly decreased cell adhesion and invasiveness in Bel7402 and SMMC cells; these effects were similar to those of sh-RNA-Twi transfection in Bel7402 and SMMC cells (p < 0.05; Fig. 3B,C).

To explore the biological significance of miR-27a-3p in VM, a well-established model *in vitro* of VM formation (3D culture) was used. The results showed that miR-27a-3p significantly decreased capillary tube formation within the 3D Matrigel medium in cultures of Bel7402 and SMMC cells, and this effect of miR-27a-3p was consistent with that of sh-RNA-Twi (Fig. 3D). Moreover, gelatin zymography was performed to detect the activity of MMP2 and MMP9, which are important for VM formation. The results showed a significant reduction in the activity of MMP2 and MMP9 in miR-27a-3p or sh-RNA-Twi-transfected Bel7402 and SMMC cells (Fig. 3E).

**Figure 4:**
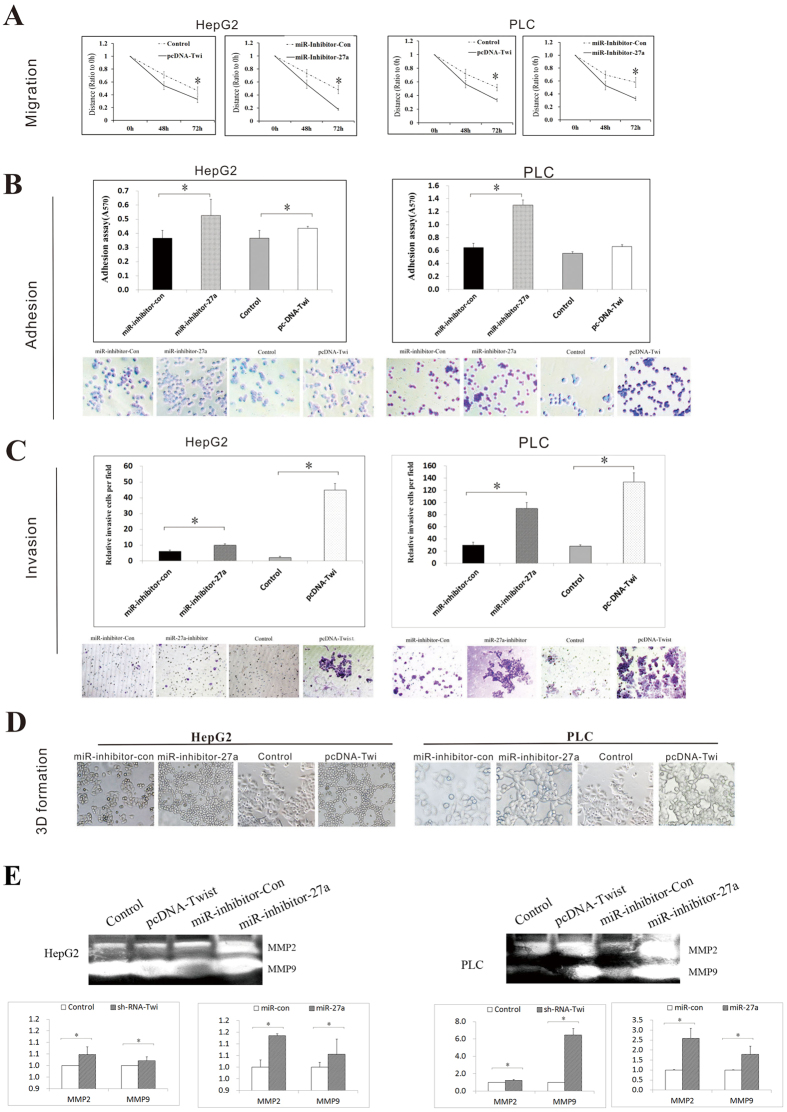
Down-regulation of miR-27a-3p enhanced aggressive phenotypes and VM formation. HepG2 and PLC cells were transfected with miArrest™ miRNA Inhibitor Expression Clone miR-27a-3p (referred to as miR-inhibitor-27a) or pcDNA3-Twist-1 (referred to as pcDNA-Twi). The effects of miR-inhibitor-27a and pcDNA3-Twist-1 on cell migration, invasion and adhesion and capillary tube formation were evaluated via (**A**) a wound healing assay, (**B**) a cell adhesion assay, (**C**) a transwell invasion assay, (**D**) a 3D VM formation assay, and (**E**) gelatin zymography.

To verify the findings in the gain-of-function model, loss-of-function experiments were performed using miArrest™ miRNA Inhibitor Expression Clone miR-27a-3p (referred to as miR-inhibitor-27a in Fig. 4) in HepG2 and PLC cells; treatment with this miRNA inhibitor clearly decreased the endogenous miR-27a-3p levels in HepG2 and PLC cells. Alternatively, pcDNA3-Twist-1 (referred to as pcDNA-Twi in Fig. 4) was transfected into HepG2 and PLC cells, which display low endogenous expression of Twist-1. Suppression of cellular miR-27a-3p expression not only promoted the invasive behavior of HCC cells but also enhanced VM formation; these effects were identical to those of Twist-1 up-regulation (Fig. 4).

Collectively, these data suggest a suppressive effect of miR-27a-3p on aggressive cellular behaviors and VM formation in cells displaying high endogenous expression of Twist-1. These results indicated that Twist-1 may induce aggressive cellular behaviors and VM formation by negatively regulating the expression of miR-27a-3p.

### miR-27a-3p targets the 3′-UTR of VE-cadherin and down regulates its expression

Next, we explored the molecular mechanisms responsible for the multiple functions of miR-27a-3p. First, potential target genes of miR-27a-3p were predicted using databases including TargetScan, PicTar, and miRanda. Among these potential targets, VE-cadherin (CDH5) was chosen for further experimental validation, not only because it was identified as a target of miR-27a-3p by all three databases, but also due to its frequent over-expression in tumor tissues and its well-known importance in both tumor invasion and VM formation^20^,^21^.

**Figure 5:**
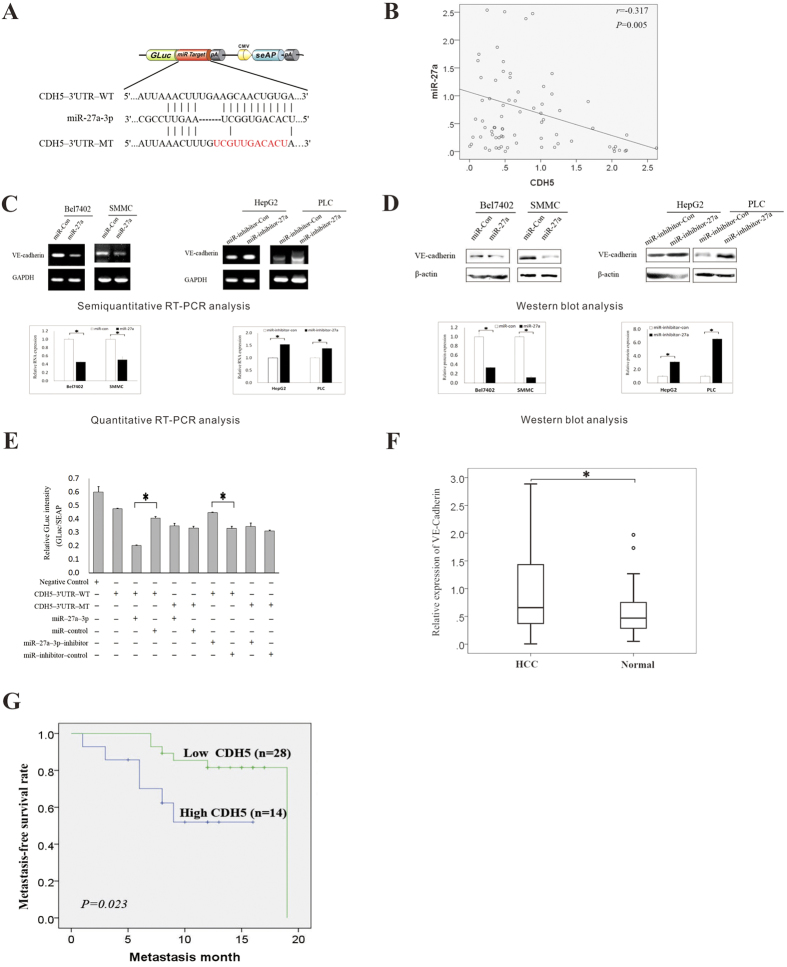
miR-27a-3p targets the 3′-UTR of the VE-cadherin gene and down-regulates VE-cadherin expression. (**A**) The sequences of the predicted miR-27a-3p binding site and the VE-cadherin (CDH5) 3′-UTR segments containing the wild-type or mutant binding site are shown. (**B**) qRT-PCR was performed to analyze the expression of miR-27a-3p and VE-cadherin in human HCC tissue. mRNA expression (**C**) and protein expression (**D**) of VE-cadherin in Bel7402 and SMMC cells transfected with miExpress™ Precursor miRNA Expression Clone miR-27a-3p (referred to as miR-27a) and in HepG2 and PLC cells transfected with miArrest™ miRNA Inhibitor Expression Clone miR-27a-3p (referred to as miR-inhibitor-27a). In immunoblotting assay, gels have been run under the same experimental conditions. Then cropped blots were incubated with different primary antibodies for analysis of signaling pathway. Full-length blots are included in the (supplementary information Fig. S5) (**E**) The relative luminescence intensities of GLuc and SEAP were analyzed after the wild-type or mutant 3′-UTR reporter plasmid was co-transfected with miR-27a-3p, miR-control, miR-27a-3p-inhibitor, or miR-inhibitor-control into 293T cells. The histogram shows the mean values ± SD of the normalized GLuc intensity (GLuc/SEAP ratio) from three independent experiments. (**F**) qRT-PCR was performed to determine the mRNA levels of VE-cadherin in clinical HCC specimens. The relative expression values are shown in scatter plots. The median expression level is indicated by a horizontal line. The VE-cadherin abundance was normalized to the abundance of U6. (**G**) Kaplan-Meier survival curves of patients with HCC indicated that higher VE-cadherin expression was significantly associated with early metastasis of HCC (*p < 0.05).

As shown in Fig. 5A, a binding site for miR-27a-3p was found in the 3′-UTR of VE-cadherin (also referred to as CDH5) at 699–722 nt. To explore the relationship between VE-cadherin and miR-27a-3p, qRT-PCR was used to analyze the expression of miR-27a-3p and VE-cadherin in human HCC tissue. Clearly, the miR-27a-3p level inversely correlated with the VE-cadherin expression level (r = −0.317, p = 0.005) (Fig. 5B). Next, Bel7402 and SMMC cells were transfected with the miExpress™ Precursor miRNA Expression Clone miR-27a-3p (denoted as miR-27a in Fig. 5), and HepG2 and PLC cells were transfected with the miArrest™ miRNA Inhibitor Expression Clone miR-27a-3p (named as miR-inhibitor-27a in Fig. 5). Semi-quantitative RT-PCR, quantitative RT-PCR and Western blot were performed to measure the changes in VE-cadherin expression. The results showed that miR-27a-3p decreased VE-cadherin expression in Bel7402 and SMMC cells but that miR-inhibitor-27a increased VE-cadherin expression in HepG2 and PLC cells (Fig. 5C,D).

To further assess whether the 3′-UTR of VE-cadherin is a functional target of miR-27a-3p, we cloned a reporter plasmid containing the wild-type (WT) 3′-UTR of VE-cadherin at the 3′ position of the Gluc/SeAP reporter gene. In parallel, we cloned another reporter plasmid in which the conserved target sequence within nt699–722 was specifically mutated (MUT); this mutation was predicted to reduce miR-27a-3p binding activity at nt699–722 (Fig. 5A). Then, a Secrete-Pair^TM^ Dual Luminescence Assay Kit was used to investigate the interaction between miR-27a-3p and VE-cadherin. The results showed that luciferase activity was markedly diminished in cells transfected with the miR-27a-3p and WT 3′-UTR reporter plasmids compared to cells transfected with the miR-27a-3p and MUT3′-UTR reporter plasmids (Fig. 5E, p < 0.05). Taken together, these data imply that miR-27a-3p attenuates the expression of VE-cadherin by directly targeting the VE-cadherin 3′-UTR.

We further analyzed VE-cadherin expression in human HCC tissues and its association with metastasis. The results showed that the expression of VE-cadherin was higher in HCC tissues than in noncancerous liver tissues (Fig. 5F) and that VE-cadherin expression correlated with early metastasis (Fig. 5G).

### miR-27a exerts its function by suppressing EMT signaling

EMT has been proposed as a key process in cancer progression in which epithelial cells acquire mesenchymal properties and exhibit increased cell-matrix adhesion and motility. There are many common pathways regulating EMT and VM. To better understand the mechanism by which miR-27a-3p inhibited metastasis and VM formation, the expression levels of EMT-associated factors were evaluated.

**Figure 6:**
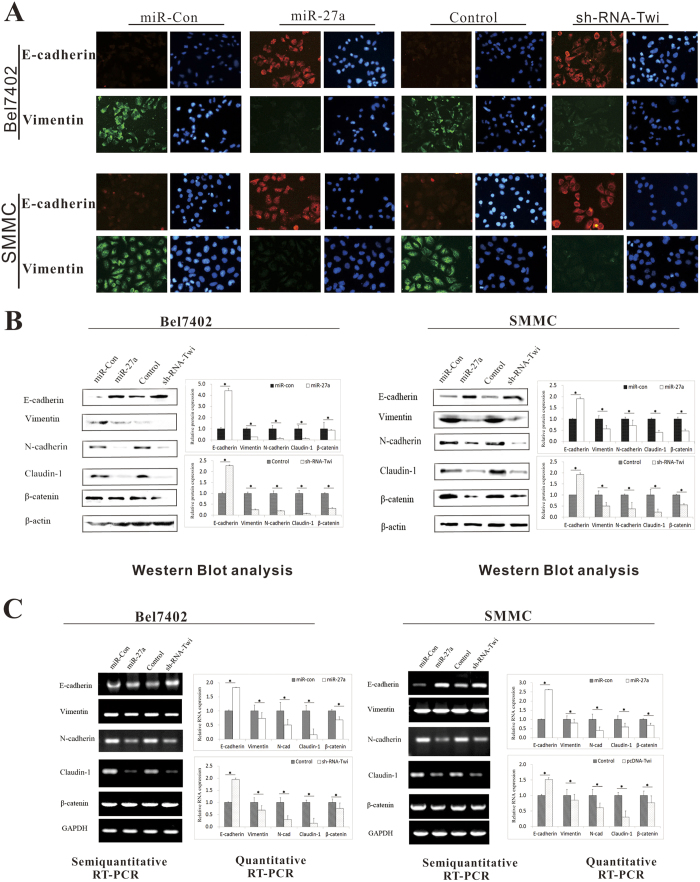
miR-27a-3p suppressed EMT signaling. Bel7402 and SMMC cells were transfected with miExpress™ Precursor miRNA Expression Clone miR-27a-3p (miR-27a) orpGP-Twist-1-shRNA (sh-RNA-Twi). (**A**) The immunofluorescence results showed the expression of E–cadherin and vimentin in Bel7402 and SMMC cells. (**B**) Western blot showed the protein expression of E–cadherin, vimentin, N-cadherin, Claudin-1 and β-catenin in Bel7402 and SMMC cells. (**C**) Semiquantitative and quantitative RT-PCR analyses showed the mRNA expression of E–cadherin, vimentin, N-cadherin, Claudin-1 and β-catenin in Bel7402 and SMMC cells. In immunoblotting assay, gels have been run under the same experimental conditions. Then cropped blots were incubated with different primary antibodies for analysis of signaling pathway. Full-length blots are included in the (supplementary information Fig. S6)

The immunofluorescence results showed that miR-27a-3p significantly enhanced the expression of the epithelial marker E-cadherin and decreased the expression of the mesenchymal marker vimentin in Bel7402 and SMMC cells transfected with the miExpress™ Precursor miRNA Expression Clone miR-27a-3p (miR-27a). These activities were consistent with the effects of transfection with pGP-Twist-1-shRNA (sh-RNA-Twi) (Fig. 6A). These changes in E-cadherin and vimentin, N-cadherin protein expression were verified by Western blot (Fig. 6B). The protein expression of β-catenin, another mesenchymal marker and claudin-1 was decreased by miR-27a-3p, as well. RT-PCR and qRT-PCR analyses revealed that transfection with miR-27a-3p also increased the E-cadherin mRNA levels and decreased the vimentin, N-cadherin, Claudin-1 and β-catenin mRNA levels compared with the control treatment (Fig. 6C).

**Figure 7:**
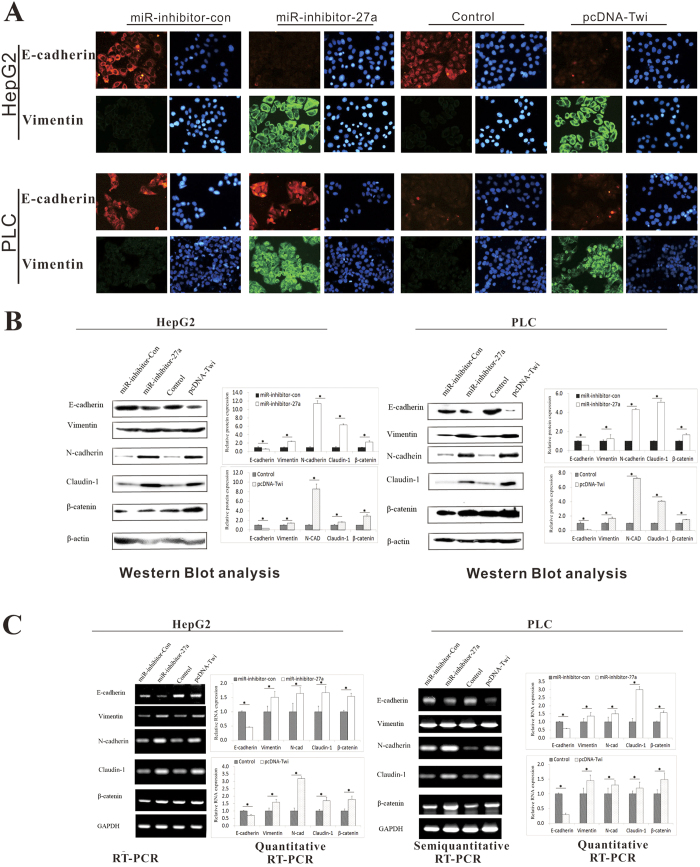
Down-regulation of miR-27a-3p induced EMT signaling. HepG2 and PLC cells were transfected with miArrest™ miRNA Inhibitor Expression Clone miR-27a-3p (miR-inhibitor-27a) or pcDNA3-Twist-1 (pcDNA-Twi). (**A**) The immunofluorescence results showed the expression of E–cadherin and vimentin in HepG2 and PLC cells. (**B**) Western blot showed the protein expression of E–cadherin, vimentin, N-cadherin, Claudin-1 and β-catenin in HepG2 and PLC cells. (**C**) Semi-quantitative and quantitative RT-PCR analyses showed the mRNA expression of E–cadherin, vimentin, N-cadherin, Claudin-1and β-catenin in HepG2 and PLC cells. In immunoblotting assay, gels have been run under the same experimental conditions. Then cropped blots were incubated with different primary antibodies for analysis of signaling pathway. Full-length blots are included in the (supplementary information Fig. S7)

Conversely, E-cadherin expression was suppressed but vimentin, N-cadherin, Claudin-1 and β-catenin expression was enhanced in HepG2 and PLC cells transfected with miArrest™ miRNA Inhibitor Expression Clone miR-27a-3p (miR-inhibitor-27a) or pcDNA3-Twist-1 (pcDNA-Twi) (Fig. 7A–C).

Taken together, our results showed that miR-27a-3p can inhibit EMT, suggesting that miR-27a-3p suppressed tumor VM and metastasis by regulating EMT.

### miR-27a-3p inhibits metastasis and VM formation by regulating EMT *in vivo*

To validate the function of miR-27a-3p *in vivo*, SMMC cells stably transfected with miR-27a-3p and HepG2 cells stably transfected with the miR-27a-3p inhibitor were subcutaneously injected into BALB/c-nu/nu mice.

**Figure 8:**
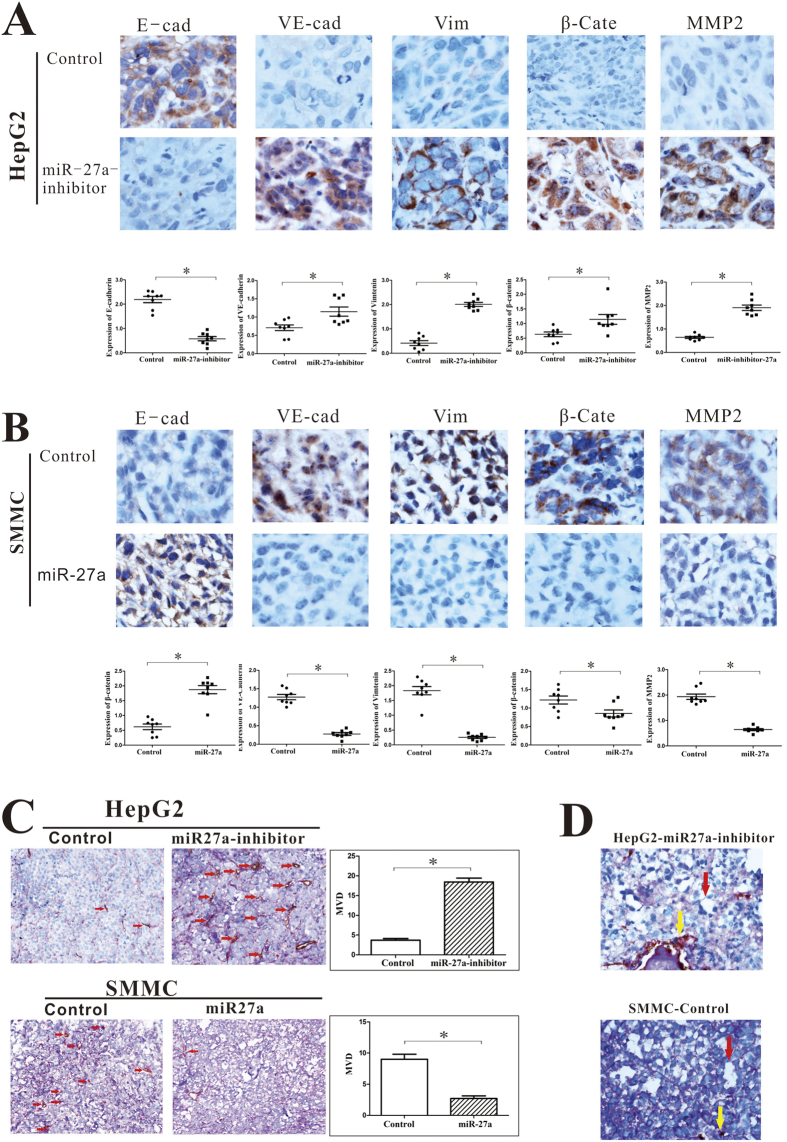
miR-27a-3p inhibits VM formation and metastasis by regulating EMT *in vivo*. HepG2 cells stably transfected with the miR-27a-3p inhibitor (miR-27a-inhibitor) and SMMC cells stably transfected with miR-27a-3p (miR-27a) were subcutaneously injected into BALB/c-nu/nu mice. IHC staining showed the expression of E-cadherin (E-cad), VE-cadherin (VE-cad), vimentin (Vim), β-catenin (β-cate) and MMP2 from the group transfected with the HepG2-miR-27a-inhibitor (**A**) and SMMC cells transfected with miR-27a (**B,C**) Endomucin/PAS double staining showed MVD based on endomucin staining (red arrows indicate MVD) (**D**). Endomucin/PAS double staining showed VM formation in HCC tissue (red arrows indicate capillary tubules formed by tumor cells; yellow arrows indicate endothelium-dependent vessels).

Consistent with our previous observation, the results of immunohistochemical (IHC) staining showed that down-regulation of miR-27a-3p resulted in low expression of E-cadherin and high expression of vimentin and β-catenin in HepG2 cells (Fig. 8A) but that up-regulation of miR-27a-3p (miR-27a) significantly enhanced the expression of E-cadherin and suppressed the expression of vimentin and β-catenin in SMMC cells (Fig. 8B). These results suggested that miR-27a-3p inhibited EMT *in vivo*.

Moreover, miR-27a-3p suppressed the expression of VE-cadherin, which is important for VM, and suppressed the expression of MMP2, which plays an important role in metastasis (Fig. 8A,B).

Furthermore, compared with the control group, the group of HepG2 cells stably transfected with the miR-27a-3p inhibitor displayed much higher MVD and the group of SMMC cells stably transfected with miR-27a-3p showed much lower MVD (Fig. 8C). Additionally, we observed VM in tissues from mice injected with HepG2 cells stably transfected with the miR-27a-3p inhibitor or with SMMC control cells; this finding suggested that miR-27a-3p inhibited the formation of VM *in vivo*, and this effect of miR-27a-3p was consistent with its effect *in vitro*.

Collectively, these findings indicate that miR-27a-3p suppresses VM and metastasis by regulating EMT *in vivo*.

## Discussion

VM was described in 1999 by Maniotis *et al.* as a process in which aggressive melanoma cells may generate vascular channels that facilitate tumor perfusion independent of endothelial cells^22^. Previous work by our group showed that the EMT regulatory factor Twist-1 might induce VM formation in HCC by down-regulating E-cadherin and up-regulating VE-cadherin^17^. Twist-1 functions via different signaling pathways, including signal transducer and activator of transcription 3, MAPK, TGFβ, and Wnt signaling^2^. A recent report identified miRNAs as contributors to Twist-1 functions in tumor metastasis and VM formation and supported a role of these miRNAs as novel intermediates in the pathways controlling the development of specific cell populations^4^. Many studies have shown that the expression of miRNAs was deregulated in various types of human cancer and that miRNAs may play essential roles in multiple biological processes, including cell differentiation, proliferation, angiogenesis, invasion, and migration. However, little is known about the relationship between Twist-1 and miRNAs and the function of miRNAs in metastasis, invasion, and VM in HCC.

Our data showed that Twist-1 up-regulation in HCC was associated with the differential expression of 18 miRNAs. Among these miRNAs, miR-27a-3p has commonly been implicated in cancer biology; interestingly, the regulation patterns and the functions of miR-27a-3p varied depending on the cancer type. In the present study, we demonstrated that miR-27a-3p functions as a tumor suppressor against metastasis, invasion, and VM in HCC by negative regulating the expression of VE-cadherin and EMT-associated markers in cancer cells.

Microarray and ChIP-seq analyses showed that miR-27a-3p may directly bind to Twist-1 and that miR-27a-3p was consistently down-regulated in HCC cells displaying high expression of Twist-1. Results of ChIP–qPCR proved that Twist-1 could directly bind to the promoter region of miR-27a-3p. Furthermore, our data demonstrated that miR-27a-3p expression was significantly down-regulated in HCC tissues compared to adjacent noncancerous tissues but that Twist-1 expression was up-regulated in HCC tissues compared to adjacent noncancerous tissues. Additionally, the endogenous expression levels of miR-27a-3p and Twist-1 were negatively correlated in HCC cell lines. The inverse expression pattern between miR-27a-3p and Twist-1 in HCC indicated a potential interaction between them. It is reasonable to propose that Twist-1 regulates miR-27a-3p expression to promote metastasis, invasion, and VM in HCC.

In previous studies, miR-27a-3p was reported to act as an oncogene in several human cancer types. For example, it was demonstrated that the inhibition of miR-27a suppressed pancreatic cancer cell growth, colony formation and migration by targeting Sprouty2^23^. High miR-27a expression has been associated with poor overall survival in patients with breast cancer^24^. In glioma, ectopic expression of miR-27a-3p promotes glioma cell proliferation via the cooperative regulation of MXI1^25^. Conversely, miR-27a-3p was reported to act as a tumor suppressor in other types of cancer. One study of ESCC revealed that miR-27a attenuated tumor proliferation, invasion and growth^14^. In addition to its involvement in ESCC, miR-27a acted as a tumor suppressor in colorectal carcinogenesis and progression by targeting SGPP1 and Smad2^15^. In another study, miR-27a was identified to suppress the clonogenic growth and migration of human glioblastoma multiforme cells by targeting BTG2^26^. In HCC, low expression of miR-27a-3p was found in plasma from patients with HCC compared with those from control subjects^27^. In additional studies, decreased miR-27a-3p expression was observed in HCC tissue compared with non-tumorous tissues^28^,^29^, and this finding suggested that miR-27a-3p functions as a tumor suppressor.

Our data indicated that miR-27a-3p expression was significantly down-regulated in patients with HCC and that miR-27a-3p down-regulation was associated with metastasis. Similarly, our gain- and loss-of-function assays showed that miR-27a-3p suppressed metastasis, adhesion, invasion, VM formation, and both MMP2 and MMP9 activity *in vitro*. The results of our *in vivo* experiments further demonstrated that miR-27a-3p reduced the aggressive behavioral phenotypes of tumor cells and VM formation. These findings suggested the tumor suppressive function of miR-27a-3p in HCC.

Regarding the tumor suppressive function of miR-27a-3p, it was reported that miR-27a-3p directly or indirectly regulates the expression of specific proteins associated with distinct stages of cancer, such as proliferation, apoptosis evasion, migration, and invasion^14^,^15^,^30^. Recently, miR-27a was identified to target the epidermal growth factor receptor^31^,^32^, which promotes tumorigenesis in numerous types of cancer. Our results revealed that ectopic expression of miR-27a-3p resulted in suppression of VE-cadherin (CDH5) expression in HCC cells but that down-regulation of miR-27a-3p resulted in increased expression of VE-cadherin. Bioinformatics tools predicted that VE-cadherin is a putative target gene of miR-27a-3p. Based on a dual-luciferase reporter assay, we confirmed that miR-27a-3p directly bound to the 3′-UTR of VE-cadherin and reduced the mRNA and protein expression levels of VE-cadherin. In breast cancer, VE-cadherin was shown to promote tumor cell proliferation and invasion by enhancing TGF-β signaling^20^. Additionally, a previous study has demonstrated that VE-cadherin is critical for VM formation in various tumors, including HCC^17^, melanoma^33^, and esophageal carcinoma^34^. It is reasonable to propose that miR-27a-3p suppresses tumor metastasis, invasion, and VM formation in HCC by targeting VE-cadherin.

EMT, which plays an important role in embryogenesis, wound healing, organ fibrosis, and cancer metastasis, is closely associated with the migration and remodeling of vascular endothelial cells^35^. Upon EMT, tumor cells acquire features that enable their progression; in particular, these properties are related to cytoskeletal remodeling, motility, and invasiveness. To comprehensively understand the effect of miR-27a-3p on cancer cells, we evaluated the expression of EMT markers in cells transfected with miR-27a-3p or miR-inhibitor-27a-3p. The results showed that high expression of miR-27a-3p significantly enhanced the expression of the epithelial marker E-cadherin and decreased the expression of the mesenchymal marker vimentin and N-cadherin in Bel7402 and SMMC cells. Downregulation of miR-27a-3p significantly suppressed E-cadherin but induced vimentin, N-cadherin and β-catenin expression in HepG2 and PLC cells. Early research predominantly identified claudins as tumor suppressors in human malignancies. Recently, a novel claudin-1 high expression group of mammary carcinomas has been identified, attributing a dual, tumor suppressor and oncogenic role in breast cancer^36^. More recently, Claudin-1 was identified to induce EMT in HCC^37^,^38^,^39^. In the present study, Claudin-1 expression showed the same trends with vimentin and N-cadherin, suggesting its role in promoting EMT may be suppressed by miR-27a-3p. These results supported the hypothesis that miR-27a-3p reverses EMT to inhibit cell migration, adhesion, and capillary tubule formation. Interestingly, based on our predictive analysis of putative miR-27a-3p targets using databases, 244 genes were identified as possible targets of miR-27a-3p. However, E-cadherin, vimentin, N-cadherin, Claudin-1 and β-catenin were not identified as miR-27a-3p target genes. Thus, the suppressive effect of miR-27a-3p on EMT-related molecules appears to be indirect. Animal models showed that miR-27a-3p significantly reduced aggressive behavioral phenotypes and VM in mice by regulating the expression of VE-cadherin and EMT markers. These results validated the function of miR-27a-3p *in vivo*.

Taken together, these results indicated that miR-27a-3p acts as a tumor suppressive miRNA in human HCC by suppressing VE-cadherin expression and EMT. These findings provide additional evidence supporting a critical role of miRNAs in HCC invasion, metastasis, and VM. Given that miR-27a-3p is down-regulated in HCC cells displaying high expression of Twist-1, miR-27a-3p may function as an essential mediator of Twist-1 in HCC. The introduction of this mature miRNA into tumor tissue could serve as a therapeutic strategy by reducing the expression of its target genes. Our findings are encouraging and suggest that this miRNA could be targeted for the future development of a treatment for patients with HCC, especially HCC exhibiting VM.

## Additional Information

**How to cite this article**: Zhao, N. *et al.* miR-27a-3p suppresses tumor metastasis and VM by down-regulating VE-cadherin expression and inhibiting EMT: an essential role for Twist-1 in HCC. *Sci. Rep.* **6**, 23091; doi: 10.1038/srep23091 (2016).

## Supplementary Material

Supplementary Information

## Figures and Tables

**Table 1:** The list of miRNAs identified in Twist-1 overexpressed HCC.

Reporter Name	Control mean	p-Twist-1 mean	*p-value*	Log2 (G2/G1)
Downregulated microRNAs				
hsa-miR-140-3p	430	104	4.57E–03	−2.05
hsa-miR-19a	46	14	2.59E–03	−1.74
hsa-miR-19b	471	163	6.00E–03	−1.53
hsa-miR-1246	1,160	483	4.42E–03	−1.26
hsa-miR-1260b	4,939	2,380	1.90E–03	−1.05
hsa-miR-486-3p	35	20	9.16E–03	−0.85
hsa-miR-9*	89	60	5.80E–04	−0.56
hsa-miR-378	436	306	8.07E–03	−0.51
hsa-miR-26b	918	651	7.33E–03	−0.50
hsa-miR-17	4,304	3,508	1.06E–03	−0.29
hsa-miR-27a-3p	9,778	8,192	5.02E–03	−0.26
hsa-miR-23b	20,418	17,754	3.66E–03	−0.20
hsa-miR-25	5,360	4,692	5.20E–03	−0.19
Upregulated microRNAs				
hsa-miR-26a	4,788	5,482	8.44E–03	0.20
hsa-miR-3195	1,342	1,543	9.70E–03	0.20
hsa-miR-151-5p	1,728	2,015	6.78E–03	0.22
hsa-miR-128	1,015	1,580	2.12E–03	0.64
hsa-miR-132	519	848	6.77E–03	0.71

The list of miRNAs identified in HepG2-Control and HepG2-Twist1with their mean expression values determined following global normalization and statistical analysis using student’st-test. Fold increase in HepG2-Twist 1 compared to HepG2-Control is shown. The P-values for each gene are <0.05.

**Table 2:** Relationship between clinicopathologic characteristics and metastasis.

Variable	Category	Available No. for Survival (N = 42)	Median month of Metastasis	*χ*^2^	*Log-rank P*-value
Age (years)	≤50	14	14.27	0.223	0.637
	>50	29	15.27		
Sex	Male	7	12.14	1.133	0.287
	Female	35	16.18		
Tumor size (cm)	≤5	29	16.97	7.195	0.007^a^
	>5	13	11.83		
Differentiation	I + II	10	10.6	5.398	0.020^a^
	III + IV	32	16.695		
Cirrhosis	Present	23	14.437	0.01	0.921
	Absent	19	15.378		

^a^Statistically significant.
