# Supplementary material for: miR-27a-3p suppresses tumor metastasis and VM by down-regulating VE-cadherin expression and inhibiting EMT: an essential role for Twist-1 in HCC
Source: Sci Rep. 2016 Mar 16;6:23091. doi: 10.1038/srep23091 (PMC4793289; doi:10.1038/srep23091)
Supplement: Supplementary Information [file srep23091-s1.pdf]

**miR-27a-3p suppresses tumor metastasis and VM by down-regulating VE-cadherin expression and inhibiting EMT: an essential role for Twist-1 in HCC**

Nan Zhao[1,2], Huizhi Sun[1], Baocun Sun[1,2,3,*],Dongwang Zhu[4], Xiulan Zhao[1,2], Yong Wang[1], Qiang Gu[1,2], Xueyi Dong[1,2], Fang Liu[1,2]， Yanhui Zhang[3]， Xiao Li[1]

1. Department of Pathology, Tianjin Medical University, Tianjin 300070, China
2. Department of Pathology, General Hospital of Tianjin Medical University, Tianjin 300052, China
3. Department of Pathology, Cancer Hospital of Tianjin Medical University, Tianjin 300060, China
4.Stomatological Hospital, Tianjin Medical University, Tianjin 300070, China

*Corresponding author: Prof. Baocun Sun, Department of Pathology, Tianjin Medical University, Tianjin 300070, PR China; E-mail: sunbaocun@aliyun.com Tel: 86-13602111192 Fax: 86-22-83336813

Table. S1 Primers for qRT-PCR analysis

| Gene | Forward primer (5'-3') | Reverse primer (5'-3') |
|---|---|---|
| Hsa-miR-27a-3p | Order from GeneCopoeia: HmiRQP0359 | Universal qPCR Primer (GeneCopoeia) |
| Hsa-miR-17-5p | Order from GeneCopoeia: HmiRQP0230 | Universal qPCR Primer (GeneCopoeia) |
| Hsa-miR-1246 | Order from GeneCopoeia: HmiRQP0078 | Universal qPCR Primer (GeneCopoeia) |
| Hsa-miR-128 | Order from GeneCopoeia: HmiRQP0125 | Universal qPCR Primer (GeneCopoeia) |
| U6 | Order from GeneCopoeia:HmiRQP0356 | Universal qPCR Primer (GeneCopoeia) |
| CDH1 | GAGTGCCAACTGGACCATTCAGTA | AGTCACCCACCTCTAAGGCCATC |
| VIM | TGACATTGAGATTGCCACCTACAG | TCAACCGTCTTAATCAGAAGTGTCC |
| CDH5 | CCTGACTGTGGAGGCCAAAGA | TTCTCACACACTTTGGGCTGGTAG |
| TWIST1 | GGCACCATCCTCACACCTCT | GCTGATTGGCACGACCTCT |
| CDH2 | CCGGAGAACAGTCTCCAACTC | CCCACAAAGAGCAGCAGTC |
| CLDN1 | AGATGAGGATGGCTGTCATTGG | CATGCTGTGGCAGCTAAAATAGC |
| GAPDH | GCACCGTCAAGGCTGAGAAC | TGGTGAAGACGCCAGTGGA |

Table.S2 Antibodies for Westernblot and Immunohistochemical Staining

| Antibody | Source | Concentration | Lot | Manufacture |
|---|---|---|---|---|
| E-cadherin | Mouse | 1:50 | Ab1416 | Abcam |
| Vimentin | Rabbit | 1:200 | ZA-0511 | ZSCB-BIO |
| VE-cadherin | Rabbit | 1:100 | Ab33168 | Abcam |
| β-catenin | Rabbit | 1:200 | Ab32572 | Abcam |
| MMP2 | Rabbit | 1:200 | 10373-2-AP | Proteintech |
| β-actin | Rabbit | 1:1000 | Ab8227 | Abcam |
| N-Cadherin | Rabbit | 1:1000 | AF4039 | Affinity |
| Claudin-1 | Rabbit | 1:2000 | AF0127 | Affinity |
| Twist1 | Rabbit | 1:50 | Sc-15393 | SANTA CRUZ |

Table. S3 Primers used for ChIP- qRT-PCR analysis

| | Forward primer (5'-3') | Reverse primer (5'-3') |
|---|---|---|
| Primer 1 | CCTCCTCCCATCTGATTTCAAG | GGGCAGGTCTCACTATGTTG |
| Primer 2 | GCTCCACAGTTTCCCAAAG | GGGCACTTACTGAGTGACATAC |
| Primer 3 | AAATCAGCCAGGAGTGGTG | GGGTCTTGCTCTGTCACTTAG |
| Primer 4 | CTTTAGTGAGTGGAGCCTCTTT | GGTCCTCACCATGACTTATGAA |
| Primer 5 | CAGGCTGCATCAGGACAC | GGGTAGCGGTACACATACATAC |
| Primer 6 | CTTTCCCAGACCAATCACAGA | CAGGCCCTCACTGCAAA |
| Primer 7 | TACCCTGGGATTGCAGATAGA | CAGAGGCGGATGCTTTAGG |
| Primer 8 | CCATTCCTCCCGTCCAAAG | CAAGGTGGGCAGAGAGTTG |
| Primer 9 | CCAACTTTCTCCCTCCTTGTC | GACAGAGCCTTGGGCTTAAA |
| Primer 10 | CAACCATGCCAGTCTCCTT | AAAGCAATGCCCACCCA |

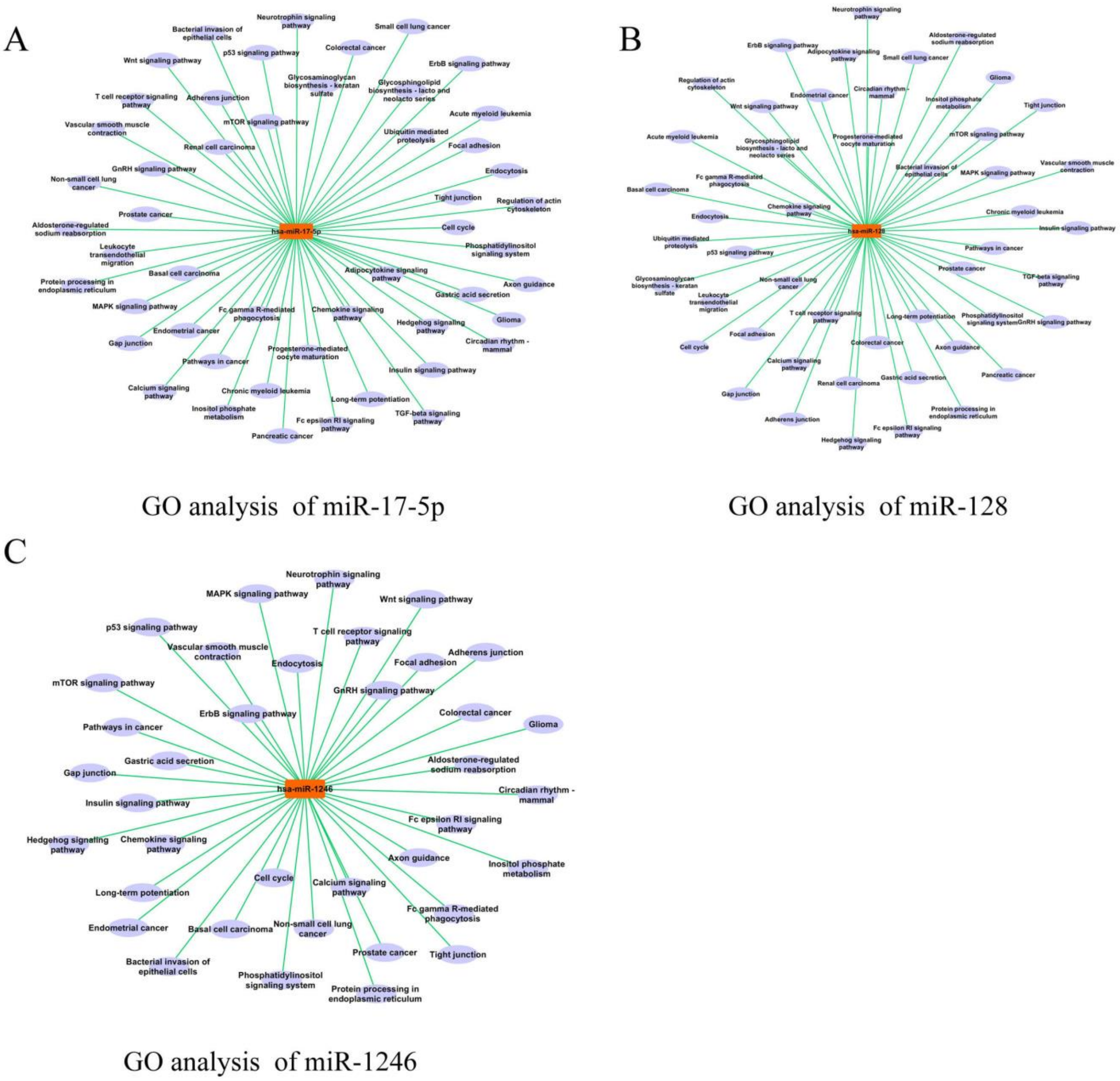


Fig. S1

(A) Microarray-based GO analysis revealed the roles of miR-17a-5p in HCC.

(B) Microarray-based GO analysis revealed the roles of miR-128 in HCC.

(C) Microarray-based GO analysis revealed the roles of miR-1246 in HCC.

**Fig. S5**

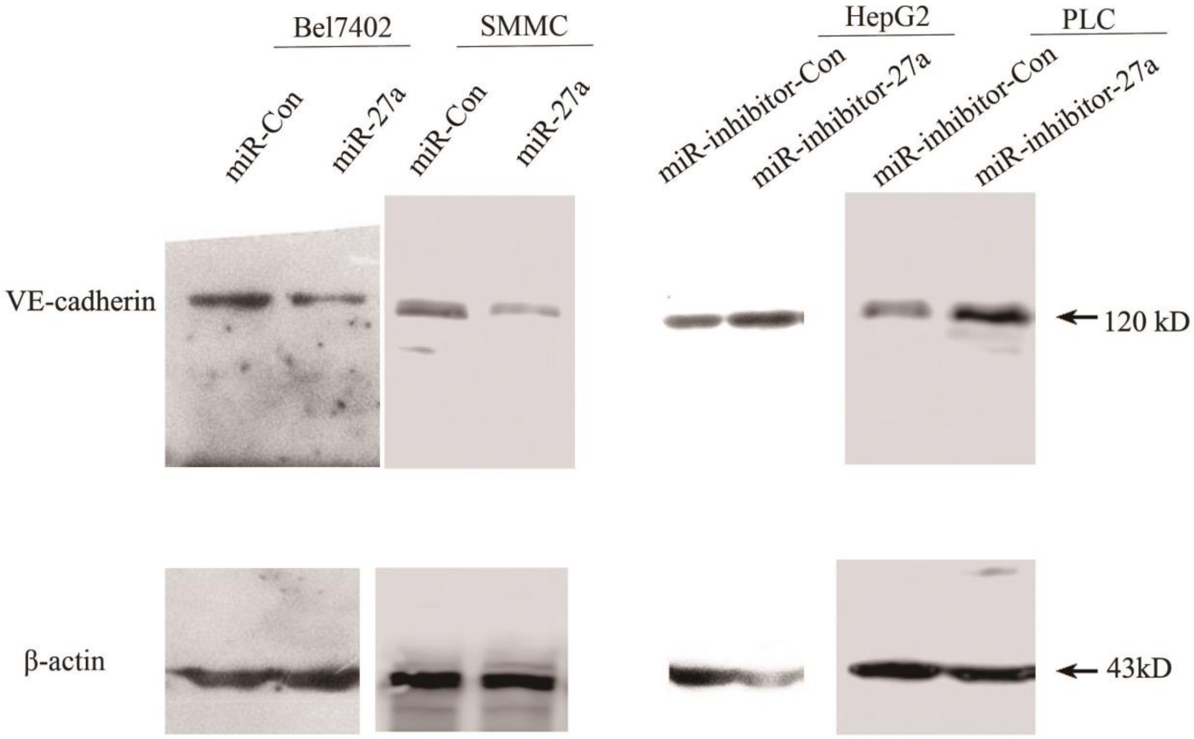

Bel7402
SMMC
HepG2
PLC
miR-Con
miR-27a
miR-Con
miR-27a
miR-inhibitor-Con
miR-inhibitor-27a
miR-inhibitor-Con
miR-inhibitor-27a
VE-cadherin
120 kD
β-actin
43kD

**Fig. S6**

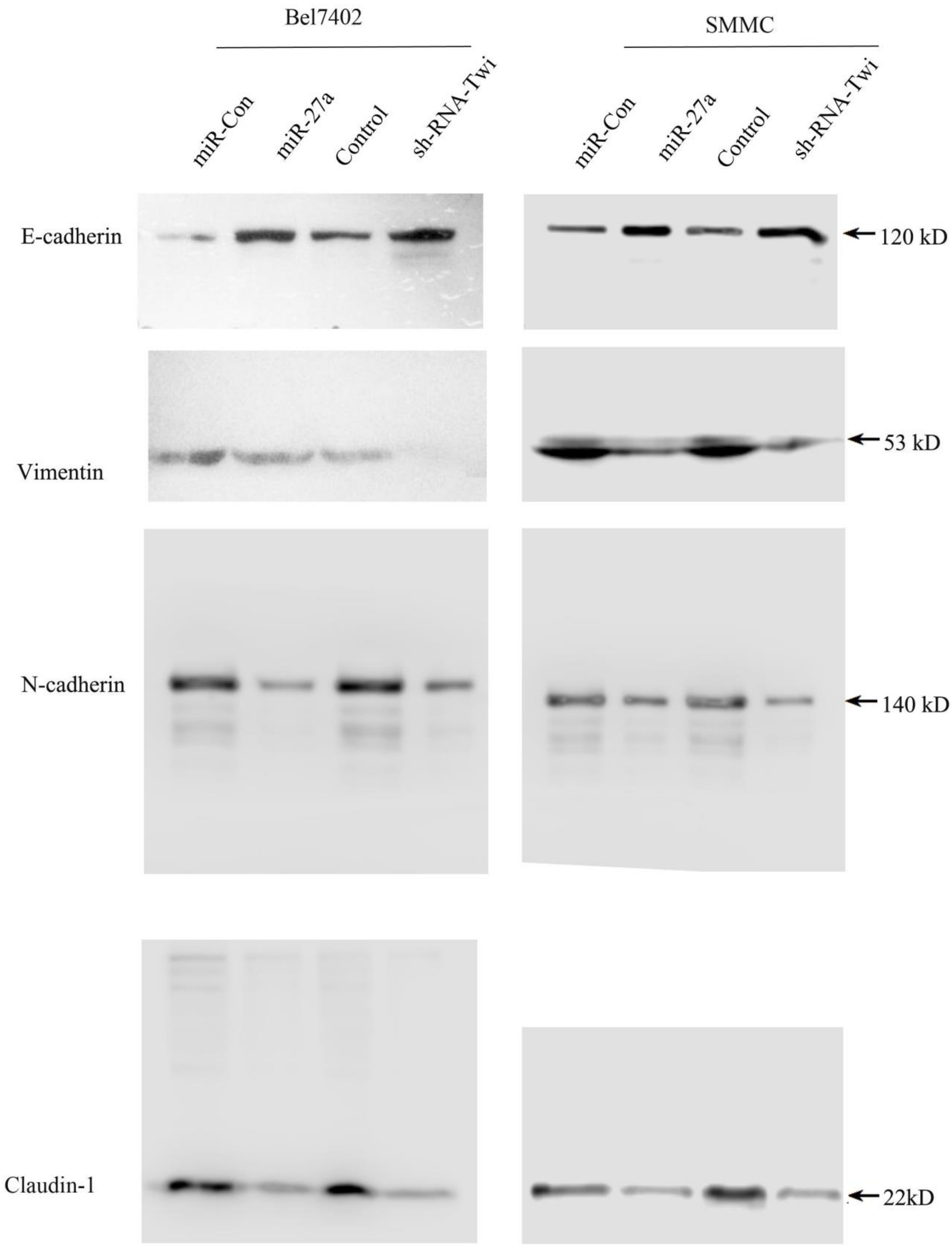
Bel7402
SMMC
miR-Con
miR-27a
Control
sh-RNA-Twi
miR-Con
miR-27a
Control
sh-RNA-Twi
E-cadherin
120 kD
Vimentin
53 kD
N-cadherin
140 kD
Claudin-1
22kD

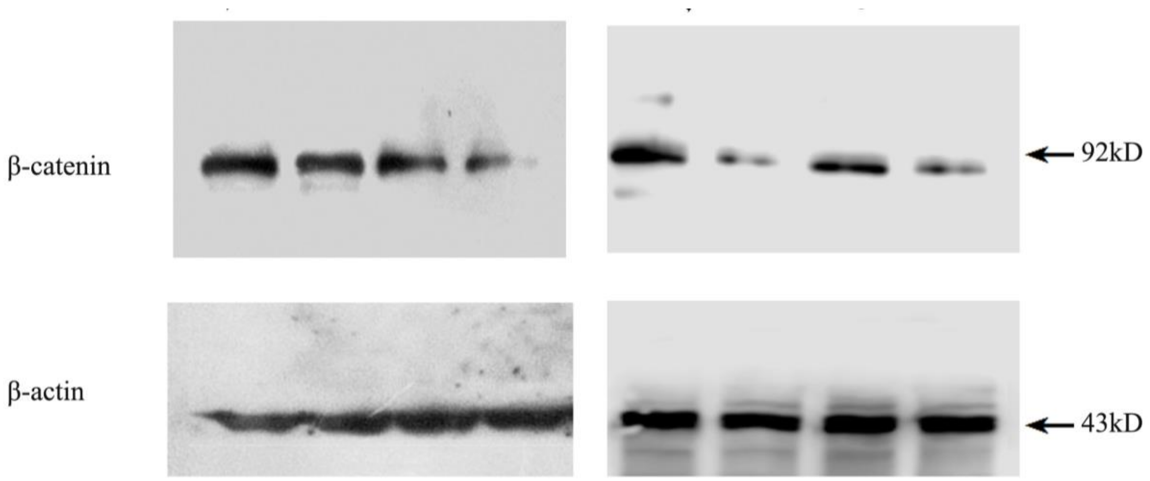

β-catenin
β-actin
92kD
43kD

**Fig. S7**

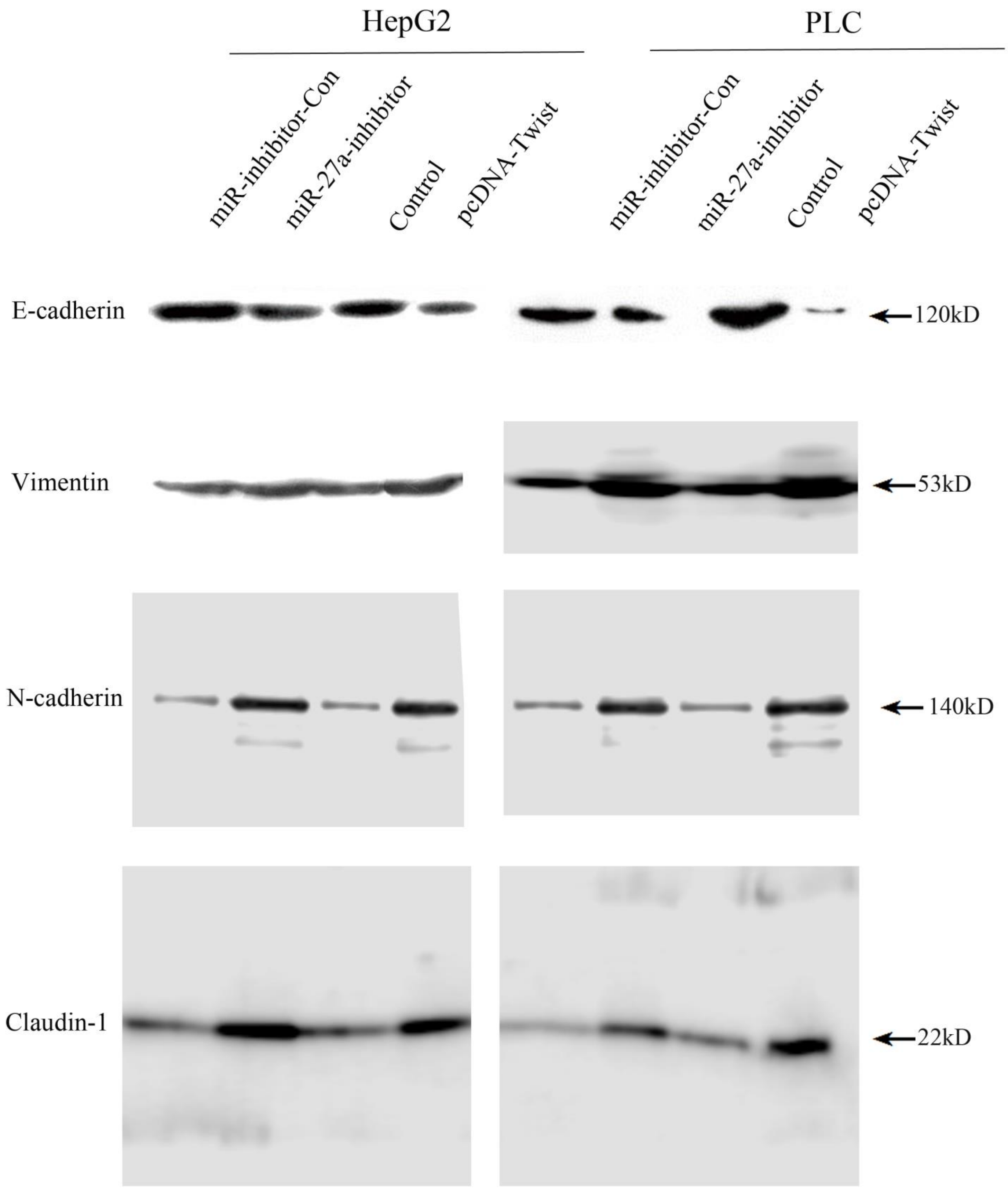
HepG2
PLC
miR-inhibitor-Con
miR-27a-inhibitor
Control
pcDNA-Twist
miR-inhibitor-Con
miR-27a-inhibitor
Control
pcDNA-Twist
E-cadherin
120kD
Vimentin
53kD
N-cadherin
140kD
Claudin-1
22kD

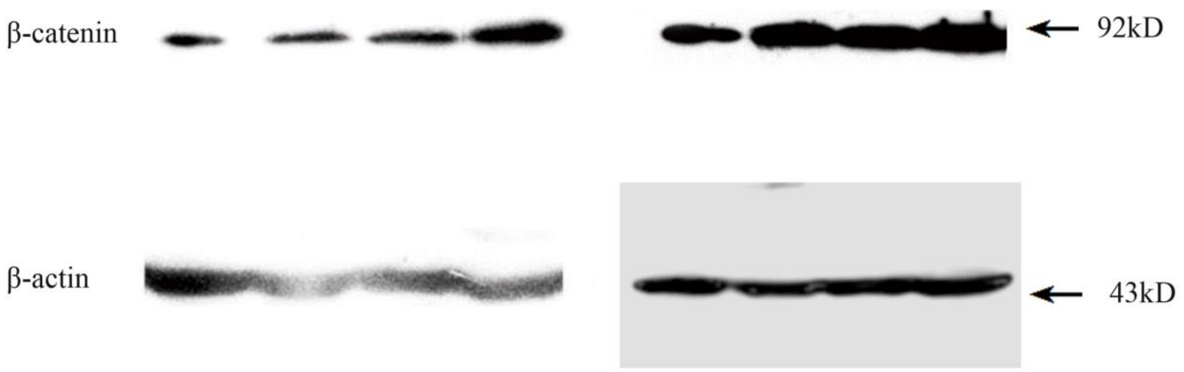
β-catenin
92kD
β-actin
43kD
